# Modulated interaction in double-layer shape memory-based micro-designed actuators

**DOI:** 10.1088/1468-6996/16/6/065003

**Published:** 2015-11-10

**Authors:** Corneliu Crăciunescu, Aurel Ercuta

**Affiliations:** 1 Politehnica University of Timisoara, RO-300006 Timisoara, Romania; 2 West University of Timisoara, RO-300223, Timisoara, Romania

**Keywords:** shape memory alloys, layered films, microactuation, modeling

## Abstract

The effect of superposed transitions in actuators with layered shape memory alloy (SMA) films undergoing martensitic phase transformation is analyzed in terms of a model developed for two layers of different composition, deposited at the same temperature on a substrate. A significant difference is observed in the actuation versus temperature relationship, depending on the thermal and elastic properties of the SMA layers and their martensitic transformation temperature. The prediction of the actuation is exemplified using a multilayer model and is verified for a cantilever actuator with NiTi and NiMnGa layers deposited on a Si substrate. The model sets the ground for a smart selection of SMAs in order to achieve a modulated actuation.

## 1. Introduction

The optimal design of shape memory alloy-based microactuators is of interest due to the very high actuation potential [1] compared to other solutions based on piezoelectric or magnetostrictive functionalities [2, 3]. Most shape memory alloy (SMA) actuators have their behavior controlled by temperature, although the magnetic control in ferromagnetic shape memory alloys is increasing their importance for practical applications [4]. The deposition of SMA films opens up a field in which the martensitic phase transformation (MPT) can be used in thinner structures, in a similar manner as in the bulk [5], but also with additional features that can improve their behavior (e.g. [6]). A steep change in the deflection vs temperature relationship occurs in bimorph actuators (i.e. SMA film deposited on a non-transforming substrate) during the martensitic transformation in the film [7, 8]. While the deflection of the free end of such a cantilever-type actuator vs temperature is linear (with a different incline when the SMA film is in the martensite and austenite state), a continuous steep slope occurs during the martensitic phase transformation, in direct relation with the changes in the fraction of martensite and austenite [9]. This is due to the fact that austenite and martensite have different thermal and mechanical properties and also to the fact that the MPT is a thermoelastic phase transformation that develops when sweeping the temperature range between *T*_MS_ (martensite start) and *T*_MF_ (martensite finish) on cooling, and between *T*_AS_ (austenite start) and *T*_AF_ (austenite finish) on heating.

Developing models for SMA-based actuator behavior for various cases allows the establishment of powerful tools that can be used to generate an actuating response based on specific inputs, such as: the architecture of the actuator (bimorph, trimorph), the specific properties of the SMA film (thermal and mechanical properties, transformation temperatures), relative thickness and sequence of the layers in layered actuators, manufacturing sequences, etc. Substantial work has been developed in modeling SMAs [10, 11] based on their thermal and mechanical properties. Thus, Zaki and Moumni [12] proposed a three-dimensional model of the thermomechanical behavior of SMAs, while Aurrichio *et al* [13] described the model for stress-induced solid phase transformation with permanent elasticity. A two-dimensional model using finite element analysis was developed by Yang and Xu [14] for bending under tip force loading of a multi-layer SMA beam. Another model, developed by Azadi *et al* [15], aimed to study the dynamic response of SMAs, also using finite element analysis. Majima *et al* [16] considered that the tracking control accuracy of SMA actuators is limited due to the inherent hysteresis nonlinearity and proposed a control system based on a static SMA model derived by modifying the classic Preisach hysteresis model. A finite element model was developed to characterize the stiffness and the damping effect, by means of an equivalent complex Young’s modulus approach under static strain offsets [17].

Compared to these approaches, our work is focused on two-way actuators with films attached to the substrate [18, 19], where models were developed based on the physical data of the films and the substrate can be used to predict the resulting actuation. This is especially important for two-way microactuators, where films usually ranging from 0.5 to 10 *μ*m deposited on metallic or nonmetallic substrates, lead to a deflection vs temperature dependence that is substantially influenced by the thermal and elastic properties of the film and the substrate, the deposition or annealing temperature of the SMA film and the temperature range for the martensitic transformation. This type of behavior can be modelled taking into consideration the above-mentioned factors of influence [20] and has important practical applications. For example, Kniknie *et al* [21] developed a thermo-mechanical model to predict the phase transformation in view of the use of SMA thin films to tune the dynamic response of atomic force microscopy micro-cantilevers. The issue can become complex for layered films, if the films are deposited at different temperatures, and could lead to a practically unlimited range of actuators, based on layers of SMAs with different compositions and/or properties.

There is an ongoing effort to define models that can be used to predict the actuation of various film-based architectures. Christophersen *et al* [22] modeled polypyrrole (PPy) bilayer microactuators and observed that there is an optimum thickness ratio if the elastic modulus of the layers is considered in the Timoshenko model [23]. Shapiro and Smela studied the bending of trilayers and suggested the use of a ‘buffer layer’ with a thickness that can be optimized so that the stress at that interface can be reduced to zero, with no loss of curvature and only a small loss in force [24].

In a previous work [20] we have developed a model that can be used to predict the actuation of bimorphs, based on differential scanning calorimetry (DSC) data of the SMA films, as well as on the elastic properties of the film and the substrate, starting from Timoshenko’s analysis of the bimetal thermostat [23]. In a subsequent work, we explored the effect of the substrate reinforcement on the actuation of shape memory alloy bimorph and trimorph architectures [25]. As the predictions fit the experimental data relatively well, it appears possible to expand the model to more complex cases, involving non-homogeneous films (i.e. multilayered films) that define a bimorph film-substrate actuator. In the current approach our aim is to develop a model that can be used to predict the behavior in architectures with SMA layers of different composition and properties (transformation temperature for this analysis) deposited on a homogeneous substrate. Since each layer has different phase transformation features, they are expected to influence the resulting deflection of a cantilever-type actuator. The model is used to predict the actuation of a cantilever based on a film containing two layers, each having its own phase transformation as well as different thermal and mechanical properties. A combination with a large difference between the phase transformation temperatures, elastic modulus and thermal expansion coefficients (e.g. NiTi and NiMnGa shape memory alloys, both known to show shape memory properties when deposited on heated substrates) was selected for the layers in order to reveal the distinct effect of each layer contribution.

## 2. Prediction of actuation in double-layered shape memory alloy films

**Figure 1.**
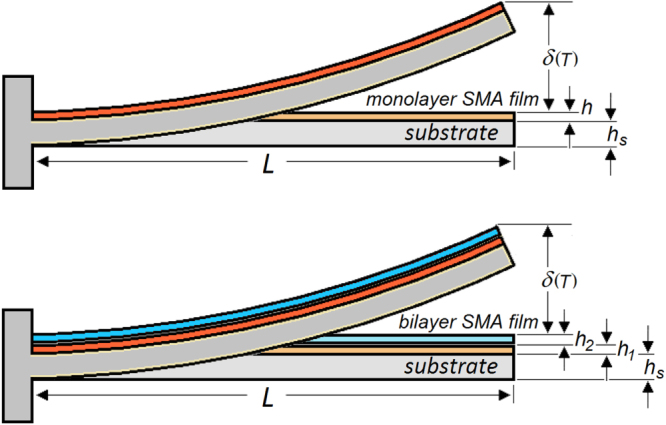
Bending on cooling from the deposition temperature *T*_D_ of a cantilever beam made of a monolayer SMA film of thickness *h* deposited onto a substrate of thickness *h*_s_ (up), and of a cantilever beam consisting of two SMA layers of thicknesses *h*_1_ and *h*_2_, deposited onto a substrate of thickness *h*_s_; in all cases, the films are assumed to exhibit thermal expansion larger than the substrate, and the beam is considered flat at *Τ*_D_.

A comparison between two cantilevers, one with a monolayer film and one with a bilayer film attached to a solid substrate (figure 1), performing thermally activated bending is discussed below, where *T* denotes temperature on the Celsius scale. In both cases, the substrate is made of a strip of length *L*, thickness *h*_s_, Young’s modulus *E*_s_, and thermal expansion coefficient *α*_s_. The monolayer SMA film is of thickness *h* and was deposited at the temperature *T*_D_ (a similar approach can be used if the film is deposited at room temperature and subsequently annealed). If the SMA undergoes MPT within the thermal range of interest,  Young’s modulus *E*(*T*) and the *α*(*T*) of the film will be temperature dependent. The bilayer film cantilever consists of two different SMA layers (say ‘layer 1’ and ‘layer 2’) of thicknesses *h*_1_ and *h*_2_, successively deposited onto an identical substrate strip; *E*_1_(*T*) and *E*_2_(*T*) are Young’s moduli, and *α*_1_(*T*) and *α*_2_(*T*) are the thermal expansion coefficients of the two SMAs, assumed to undergo MPT within different *T*_MS_–*T*_MF_ and *T*_AS_–*T*_AF_ ranges.

By neglecting the relative change in length and thickness of the components due to the thermal expansion (a calculus-justified simplification), the temperature-dependent deflection 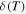 of such a cantilever beam may be expressed as

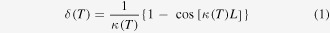
where 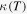 is the curvature of the beam. Based on Malzbender’s model [26] for the thermoelastic behavior of a multilayered structure, in the absence of external load this curvature may be written as

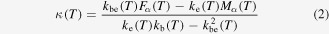
where 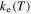 is the extensional stiffness, 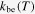 is the bending-extension coupling stiffness, and 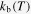 is the bending stiffness, while 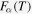 is the thermal actuation-induced force, and 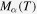 is the thermal actuation-induced moment. These quantities depend on the number of layers, their thermal and elastic properties, and alsotheir thickness. Thus, from a calculus similar to that of Du *et al* for a multilayer structure of conducting polymer films [27], the following expression

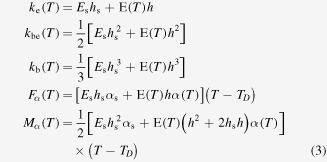
results for the monolayer-film cantilever. Concerning the thermal expansion coefficients and Young’s moduli of the SMA film, their temperature-dependence will primarily be attributed to the martensitic phase transformation. Then, by regarding the film as a mixture of two solid phases (here, martensite and austenite), it is possible to express 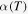 and 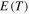 in terms of the model proposed by Hsieh [28] to describe the thermoelastic behavior of a composite material consisting of two components, locally arranged in a *serial* (*isostrain*) or in a *parallel* (*isostress*) configuration. By averaging between these two bounding approximations, the following expressions:

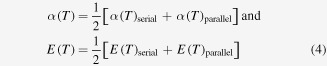
result for the SMA film, where

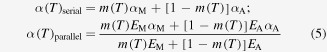
and

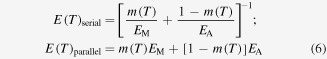
respectively. Here, 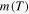 is the volume fraction of the martensite phase, while 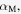 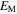 and 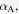 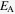 are the material constants of the martensite and austenite phases, respectively (the temperature dependence of these quantities may also be considered). If isochronal DSC (at constant heating/cooling rate 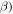 is used to determine the thermal evolution of the volume fraction of the transformed phase, the heat flow per unit mass 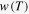 is proportional to the first derivative vs temperature of this volume fraction

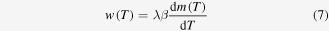
where 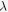 is the latent heat; note that martensite 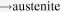 (M 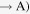 transformation is endothermal. It is then possible to obtain an analytic form for 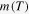 from fitting the DSC peak, provided baseline correction was operated. In this view, we have previously shown [20] that 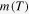 may be expressed as a weighed superposition of a finite number *N* of asymmetric sigmoid functions with weights 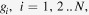 thus rewritten to include the DSC peak temperatures 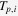 as parameters

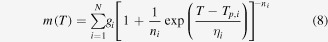
while the other two parameters 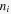 and 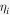 in expression (8) control the transition sharpness and scale, respectively.

Similar expressions will be considered in the case of the bilayer-film cantilever, except for the fact that the curvature (2) will now result from the following quantities

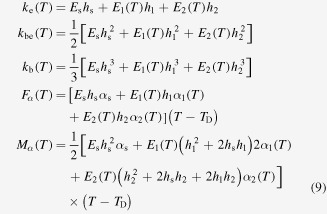
and also except for the fact that temperature-dependent Young’s moduli and thermal expansion coefficients are separately evaluated for the two SMA films (here, all the films characteristics are indexed by 1 and 2).

The model is first tested on two pairs of hypothetical monolayer film cantilevers denoted say, {I, II} and {I′, II′}, each made of a 1 *μ*m thick homogeneous SMA film deposited onto a 100 *μ*m thick and 20 mm long Si substrate beam, ( 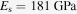 [29], 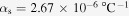 [30]); the films are assumed to exhibit MPT ( 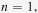 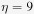 for all cases) and thermal and elastic properties as given in table 1 (one set of thermoelastic properties in the range of NiTi and the other at half values compared to the former).

**Table 1.** Thermal and elastic parameters of the hypothetical films considered in the model.

	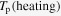 (°C)	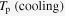 (°C)	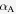 (°C^−1^)	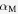 (°C^−1^)	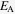 (GPa)	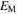 (GPa)
film I	50	0	22 × 10^−6^	13.2 × 10^−6^	160	80
film II	200	150	11 × 10^−6^	6.6 × 10^−6^	80	40
film I′	50	0	11 × 10^−6^	6.6 × 10^−6^	80	40
film II′	200	150	22 × 10^−6^	13.2 × 10^−6^	160	80

**Figure 2.**
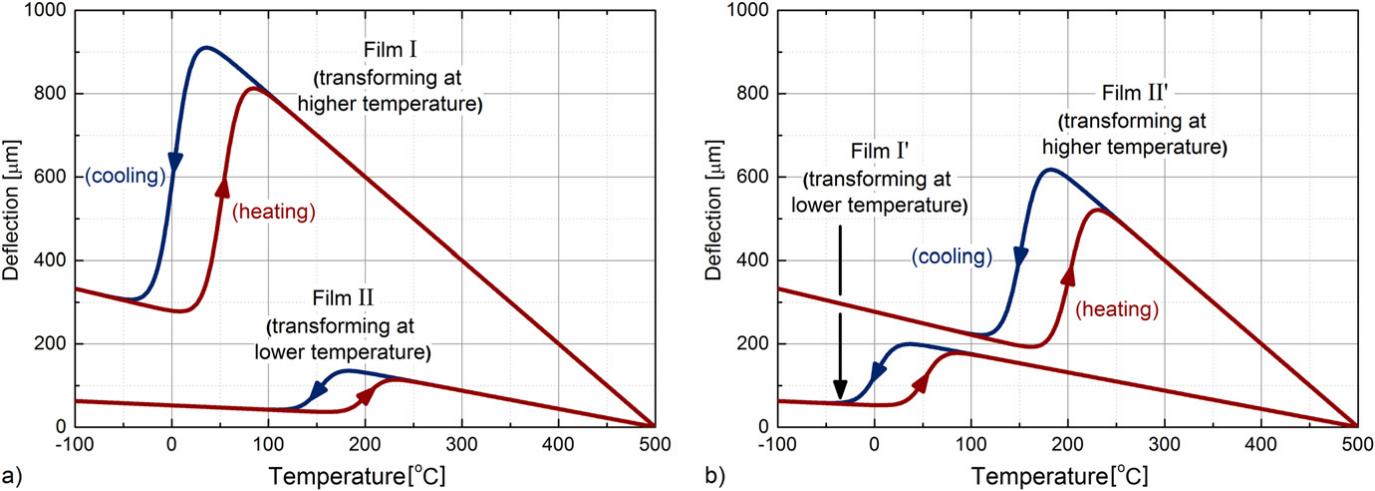
Simulation of the temperature-controlled actuation of monolayer film cantilever (one homogeneous SMA film deposited on a substrate). The films are deposited at the same temperature on Si substrates; see text for details.

The deflection of the actuators was calculated on the basis of equation (1) and the data in table 1. In figure 2 the results are plotted vs. temperature as distinct cases:

• the case in which the film that has higher differences between the elastic moduli and thermal expansion coefficients of the film and the substrate transforms at lower temperature compared to the film with lower such differences (figure 2(a)), and
• the case in which the film that has higher differences between the elastic moduli and thermal expansion coefficients of the film and the substrate transforms at higher temperature compared to the film with lower such differences (figure 2(b)).

As can be seen, the changes in the thermal and elastic properties during MPT have a strong influence on the slope of the deflection-temperature curve. For the case in which there are higher differences between the elastic moduli and thermal expansion coefficients of the film and the substrate, a higher change in the deflection is observed (films I and II’) compared to the case in which these differences are smaller (films II and I’), regardless the temperature range of the phase transformation. As expected, for monolayer film cantilevers, with the SMA layers (one transforming at a lower temperature than the other) having similar thermal and elastic properties and deposited at the same temperature and on the same type of substrate, the film that transforms at the higher temperature shows a lower actuation than the one that transforms at the lower temperature and showing higher actuation (e.g. in figures 2(a) and (b), film II transforming around 200 °C shows a lower deflection than film I’ transforming around 0 °C, and similarly film II’ shows a lower actuation than film I). This is in agreement with the experimental results reported by Winzek and Quandt [18] for annealed SMA films having different transformation temperatures, and clearly remains valid for films deposited at high temperature, usually not requiring further annealing.

**Figure 3.**
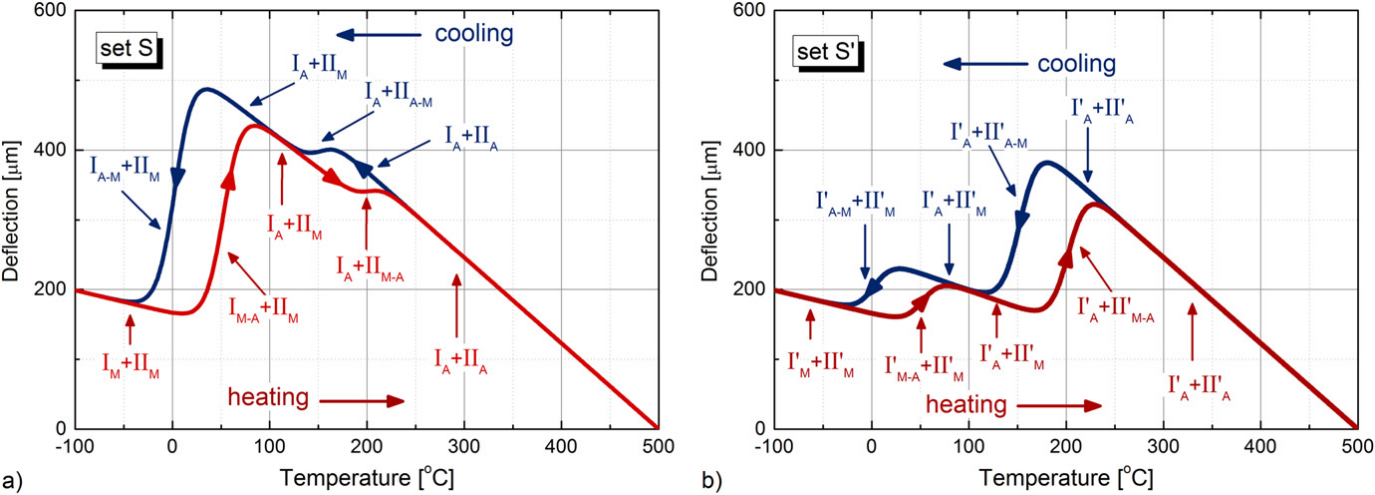
Comparative example of the predicted actuation (two layers of 0.5 *μ*m thickness each), deposited at the same temperature on a Si substrate. The two different actuators are designed with two layers as in the cases considered in figures 2(a) and (b) (zero deflection corresponds to the deposition temperature [20]). Austenite and martensite phases are denoted by A and M, respectively.

In figure 3, two cases of actuation with bilayer films resulting from combining the set of films described in figure 2(a) (sequence S in figures 3) and 2(b) (sequence S′ in figure 3), respectively, are presented.

Several distinct regions can be discerned in figure 3 for both actuators with double layer shape memory alloy films.

• The bimetal-like deflections outside the MPT ranges reflect the contribution of the austenite (I_A_ and II_A_) and martensite (I_M_ and II_M_) phases and originate in the pure thermoelastic stress as the temperature changes.
• Within the transformation range, each layer influences the deflection by additional contribution resulting from the corresponding MPT. For example, in figure 3(a), the M–A transformation in layer I on heating (I_M–A_), leads to a significant and steep increase in the deflection. By contrast, the M–A transformation in layer II on heating (II_M–A_), has only a slight hindering effect on the now decreasing deflection.
• The influence of the phase transformation temperatures of the different layers in the film appears more relevant when examining the actuations in figures 3(a) and (b); in the latter, the contributions of both layers are comparable.

It appears clear from both cases considered that the cooling path is only affected by the thermal hysteresis (and eventually by the changes in the transformation profile). This was previously discussed for monolayer film-based cantilevers on Si substrates in NiTi [31] and NiTiCu [32] systems. Accordingly, in what follows, our approach is restricted to the heating path of the actuation.

Compared to bulk polycrystalline SMAs, the properties in films can vary in an extremely large range, mainly due to the possibility to achieve a particular texture as a result of the constraints that develop at the film–substrate interface. Adding this to the range of SMAs that can be used in combinations for manufacturing the layers, the resulting actuation can vary within really large limits. This predictive tool allows a study of possible combinations that can be used to achieve the desired thermal actuation. It also worth mentioning that the elastic and thermal properties are not constant for the entire range of temperature and, when there is a large change in their temperature relationship, this needs to be accounted for. This is the case for NiMnGa SMAs, a case considered in the experimental approach.

## 3. Experimental validation of the double layer actuation concept

**Figure 4.**
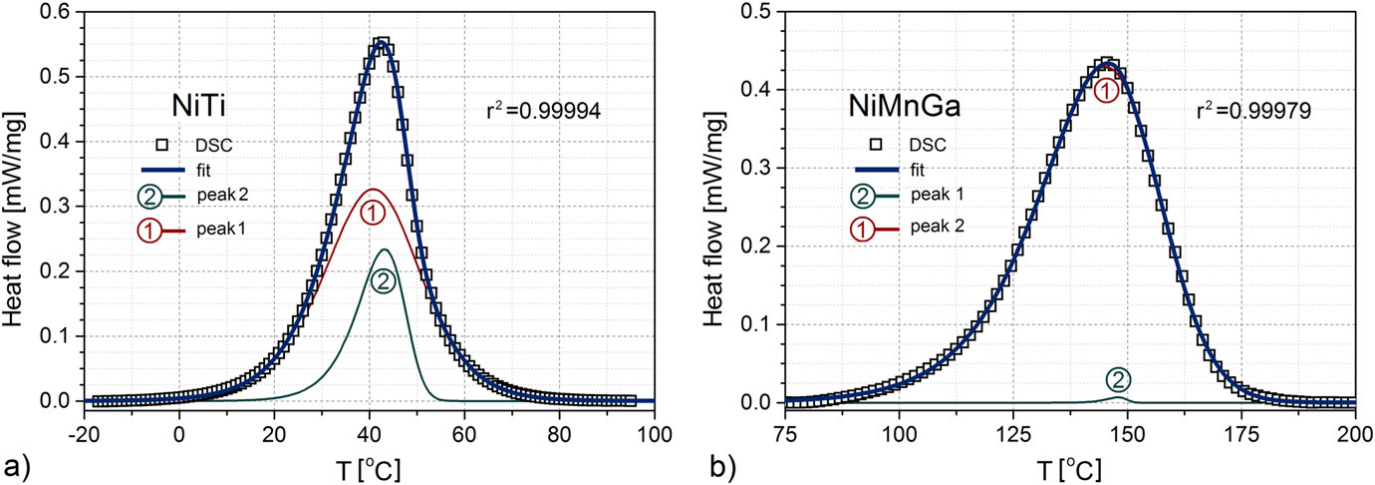
DSC profile on heating for NiTi (left) and NiMnGa (right) used in the development of the model; the coefficient of determination 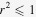 is a direct measure of the goodness of fit.

Nearly equiatomic (Ti-rich) Ni_49.93_Ti_50.07_ and Ni_48.62_Mn_33.16_Ga_18.22_ shape memory alloys were selected as target materials for the DC magnetron sputtering experiments (composition determined in a TESCAN Vega 3LM electron microscope, equipped with a Bruker Quantax 200 energy dispersive x-ray spectroscopy (EDX) system with Peltier-cooled XFlash 410M silicon drift detector). In order to determine 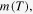 samples were extracted from the target alloys, quenched from 900 °C in water and subjected to DSC, using a DSC7 (Perkin Elmer) instrument. Parameters 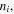 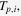 and 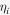 were determined from fitting the as-recorded DSC peaks (on heating), on the basis of equations (7) and (8) for *N* = 2, while the weights 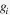 were evaluated from the corresponding subtended areas. In figure 4 the results of the fitting procedure (Lever-Marquand, least squares) are shown, and in table 2 the values of the as-resulted parameters are listed.

**Table 2.** Sigmoid parameters and weights as resulting from fitting the DSC peaks.

Material	*N*	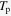 ^a^ (^o^C)	*η*^a^(^o^C)	*n*^a^	*g*
NiTi	2	40.79	7.12	1.18	0.738
		43.15	4.60	7.10	0.262
NiMnGa	2	145.12	11.22	3.03	0.999
		147.8	2.18	68.19	0.001

a As defined in equation (8).

While the thermal and elastic properties of NiTi SMAs are widely reported in the literature, and can be used to develop a model with certain accuracy [19], Young’s modulus values for NiMnGa alloys are uncertain and depend on the measurement technique and composition [33–38]. In addition, the temperature dependence of the elastic modulus apparently cannot be neglected [39]. Table 3 contains the thermal and elastic data used in the model (for NiMnGa, the thermal variation of Young’s modulus is expressed in terms of the experimental DSC peak temperature *T*_p_, based on data from [39]).

**Table 3.** Thermal and elastic data used in the model.

	Si substrate	NiTi film	NiMnGa film
Thermal expansion coefficients	2.67 · 10^−6^ °C^−1^		
• austenite		11 · 10^−6^ · °C^−1^	15 · 10^−6^ · °C^−1^
• martensite		6.6 · 10^−6^ · °C^−1^	18 · 10^−6^ · ^o^C^−1^
Young’s modulus	181 GPa		
• austenite		80 GPa	16 + 0.03(*T*–*T*_*p*_) [GPa]
• martensite		40 GPa	9-0.12 (*T*–*T*_*p*_) [GPa]

**Figure 5.**
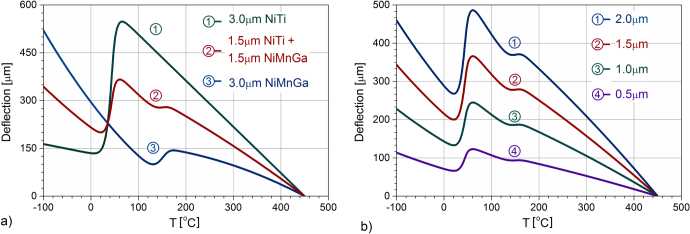
Actuation vs temperature predicted relationships on heating for actuators with SMA films deposited on 100 *μ*m Si substrate: (a) with 3.0 *μ*m monolayer films (NiTi and NiMnGa, respectively) and a 1.5 *μ*m NiTi + 1.5 *μ*m NiMnGa bilayer film, and (b) for layers of equal thickness, where 0.5 *μ*m denotes (0.5 *μ*m NiTi + 0.5 *μ*m NiMnGa), etc.

Based on the values in tables 2 and 3, functions *α* (Τ) and *E*(Τ) were determined and the deflection of the free end cantilever were calculated and plotted as a function of temperature in figure 5(a), for three distinct cases: two monolayer-type actuators (one with 3 *μ*m NiTi and one with 3 *μ*m NiMnGa films, respectively), and a double layer-type actuator (with 1.5 *μ*m NiTi film and 1.5 *μ*m NiMnGa film), all deposited on similar Si substrates (20 mm long, 100 *μ*m thick). In addition, the effect of the film thickness on the double layer actuator deflection is examined; several results are plotted in figure 5(b) for layers of equal thickness, ranging from (0.5 *μ*m NiTi + 0.5 *μ*m NiMnGa) to (2.0 *μ*m NiTi + 2.0 *μ*m NiMnGa) deposited on the same 100 *μ*m thick Si substrate.

The influence of the martensitic phase transformation in each film is reflected by the change in the corresponding actuation profile, and the way the effects combine in the double layer for the particular values considered in tables 2 and 3 is consistent with the previous predictions, as described in figures 2 and 3. Moreover, it also reveals the distinct contribution of NiTi and NiMnGa films to the resulting actuation.

In order to check the validity of the predictions on the deflection of the bilayer film-based cantilever, layers of the same thickness (1.5 *μ*m) were deposited on a pre-oxidized micro fabricated Si substrate (100 *μ*m thick), heated at the same temperature (the temperature during the deposition was 450 °C). The depositions were made under 1.3 Pa argon pressure, with the chamber being pre-evacuated to 2.7 · 10^−3^ Pa. The temperature-dependent actuation of the as-manufactured cantilever was measured capacitively in an AE-102 Acoustic Elastometer (Vibran Technology®) measurement system by recording the changes in the gap between the measuring electrode and the surface of the cantilever free-end. The record was performed on heating at 1 °C min^−1^, in a 1.3 · 10^−2^ Pa vacuum. As the elastometer is limited to floating reference, the zero deflection was adjusted in order to allow a comparison with the predicted data.

**Figure 6.**
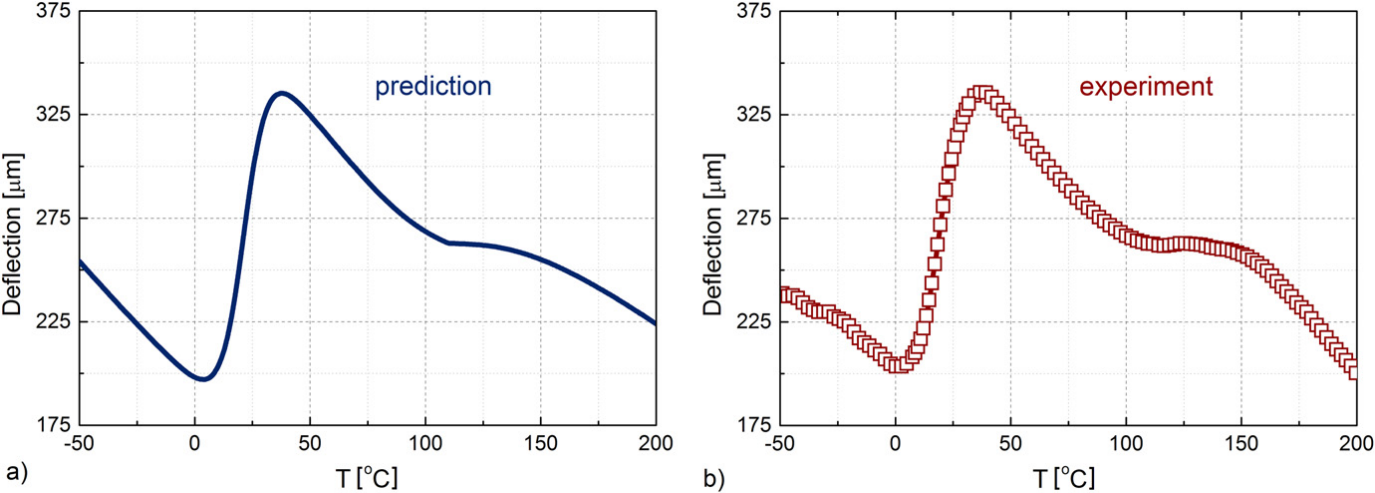
Comparison between the predicted and experimental deflection on heating during the martensitic phase transformation for a bilayer film-based actuator made of two SMA films (1.5 *μ*m NiTi and 1.5 *μ*m NiMnGa) deposited at 450 °C on a 100 *μ*m Si substrate.

Figure 6 shows a comparison between the predicted deflection (on heating) of a bilayer film-based actuator made of two SMA layers (1.5 *μ*m NiTi and 1.5 *μ*m NiMnGa) deposited at 450 °C on a 100 *μ*m Si substrate and the experimental data recorded on heating for a cantilever of the same architecture and components (i.e. films and substrate).

As figure 6 shows, there is a relatively good agreement between the experimental and predicted data. The differences can be attributed to the difficulties in precisely accounting for the thermal and elastic properties (note that the elastic moduli and thermal expansion coefficients were extracted from the literature), and especially for their eventual evolution with temperature. In addition, the slight temperature shift between the predicted and observed deflection can be attributed to the fact that the DSC data were taken out of the alloy targets and not from the films (according to Ohta *et al* [40], a reliable DSC signal requires a minimum mass of SMA samples of about 1 mg, which is significantly higher than that of the available film, e.g. for a sample of 2 mm × 5 mm surface area, this corresponds to about 15 *μ*m film thickness). Moreover, our films are attached to the substrate, which brings out additional stress influencing the transformation profile. However, the use of DSC data from the target seems to be the most adequate to be used in the predictive model since it allows estimation of the potential outputs for a large variety of actuators, prior to the manufacturing process.

## 4. Conclusions

A model has been developed for predicting the thermally activated actuation behavior of a cantilever consisting of a bilayer SMA film, deposited onto a non-transforming solid substrate. The changes in the thermoelastic properties of the SMA layers during their MPT were taken into account.

To this end, the evolution of Young’s moduli and thermal expansion coefficients of the two layers were expressed in terms of their temperature-dependent martensite phase volume fractions, using a serial/parallel model for composites. The curvature of the cantilever beam was expressed in terms of the general theory describing the multilayer thermal behavior, and the thermally activated deflection of the cantilever beam was quantified.

In the martensite and austenite state, each layer has a bimetal-like contribution to the deflection: within the thermal ranges of the reversible phase transformation, each layer influences the resulting actuation (with a more or less steep change in the deflection slope) by the corresponding changes in its thermal and elastic properties.

Predictions made for a cantilever-type actuator with a double layer NiTi/NiMnGa film deposited on a heated Si substrate show two distinct steep actuation ranges with deflection slopes associated to the MPT in each layer. With the thermal evolution of the martensite phase fraction as determined from the DSC data of the corresponding alloy targets (and thereby, that of the thermal and elastic properties), good agreement was obtained with the experimental data recorded for the actuation.

The results prove that the modulation of the thermal response for the deposition of double layer SMA films is capable of bringing out a wide range of advantages compared to the monolayer films. The resulting behavior can be tuned for the desired output by appropriately selecting the relevant properties, i.e. the elastic moduli, thermal expansion coefficients, martensitic transformation temperatures for each layer, as well as the deposition or post-deposition annealing temperature.
